# Design of Low-SAR High-Efficiency Terminal Antenna Using Magnetic Field Homogenization

**DOI:** 10.3390/mi16080856

**Published:** 2025-07-25

**Authors:** Sihan Xiao, Yong-Chang Jiao, Ziming Lv, Liupeng Zan, Zibin Weng

**Affiliations:** The National Key Laboratory of Radar Detection and Sensing, Xi’an 710071, China; 23021211267@stu.xidian.edu.cn (Z.L.); lpzan@stu.xidian.edu.cn (L.Z.); zibinweng@mail.xidian.edu.cn (Z.W.)

**Keywords:** specific absorption rate (SAR), magnetic field homogenization, high efficiency, terminal antenna

## Abstract

A low-SAR high-efficiency terminal antenna based on the magnetic field homogenization is proposed in this paper. Starting from the spatial correlation between the antenna’s near-field tangential magnetic field hotspots and SAR distribution, the influence of hotspot distribution on SAR was analyzed, and we found that a homogenized tangential magnetic field distribution can reduce the SAR without compromising the radiation efficiency. Based on the SAR reduction mechanism, a low-SAR high-efficiency terminal antenna was designed. By adjusting the magnetic field distributions on two planes with the highest initial SARs, 39% and 27% SAR reduction is achieved, respectively. Measurement results show that the antenna operates in the 3.3 GHz to 3.8 GHz band, with a radiation efficiency exceeding 69%, and a peak 10 g average SAR of 1.39 W/kg at 3.6 GHz.

## 1. Introduction

As wireless communication becomes increasingly intertwined with daily life, people are using mobile devices such as smartphones more frequently. Prolonged exposure to the electromagnetic radiation emitted by mobile phones may potentially have adverse effects on human health. To ensure that the intensity of electromagnetic radiation from mobile phones remains within safe limits, the industry has introduced the specific absorption rate (SAR) to quantify the amount of electromagnetic energy absorbed by a unit mass of biological tissue, limiting the SAR values of terminal devices such as smartphones within specified ranges [1,2,3].

To mitigate radiation hazards from terminal devices to the human body, many researchers have focused on developing low-SAR terminal antennas. The simplest way to reduce the SAR is to increase the distance between the antenna and human tissues [4,5,6]. In another popular SAR reduction method, some wave-blocking materials are employed between the devices and the human body to reduce the electromagnetic radiation toward the human body. These materials include ferrite sheets [7,8], split-ring resonators (SRRs) [9,10], and conductive materials [11], which can reflect or absorb the electromagnetic energy generated by the terminal antenna toward the human body; or the magnetic materials [12,13], the electromagnetic bandgap structures (EBGs) [14,15,16], and the high-impedance surfaces [17,18], which can suppress the surface current propagation, thus reducing the SARs. However, the prevailing design trend toward terminal devices with full-screen and ultra-thin features leaves very little space for the antennas, making these two kinds of methods impractical to apply.

In [19], H. Wang analyzed the human body electromagnetic field absorption mechanism for a mobile device antenna based on the electromagnetic field boundary conditions, and predicted the location of the maximum electromagnetic field hotspot in the human body. Building on this, a reverse current configuration to suppress the tangential electric field components in the human tissues was proposed in [20], and the designed mobile phone antenna based on the current structure distribution has low SAR characteristics. Since then, partial reverse current distributions have been used by many researchers to reduce the tangential electric fields at near distances, obtaining some low SAR antennas [21,22,23,24]. However, the introduction of partial reverse currents reduces the antenna radiation efficiencies. Therefore, a trade-off between the antenna efficiency and the SAR seems to be required, and the design of a low-SAR terminal antenna with high efficiency is necessary and urgent.

The main contributions of this paper are summarized as follows:

(1) Both simulation verification and theoretical derivation have been performed to demonstrate the correlation between the SAR and the tangential magnetic field distribution of the antenna.
(2) A magnetic field homogenization design mechanism for reducing the antenna SAR values is proposed. Unlike traditional approaches that introduce reverse currents to reduce the SAR values, this mechanism achieves homogenization of the tangential magnetic field distribution in the antenna near-field region through antenna structure optimization, and effectively reduces the SARs while avoiding degradation of antenna efficiency.
(3) Guided by the magnetic field homogenization mechanism, we design an antenna operating in the N78 band, which simultaneously achieves both high efficiency and low SAR performance.

## 2. Theoretical Analysis of Magnetic Field Homogenization

### 2.1. Tangential Magnetic Field and SAR Hot Spot Analysis

**Figure 1:**
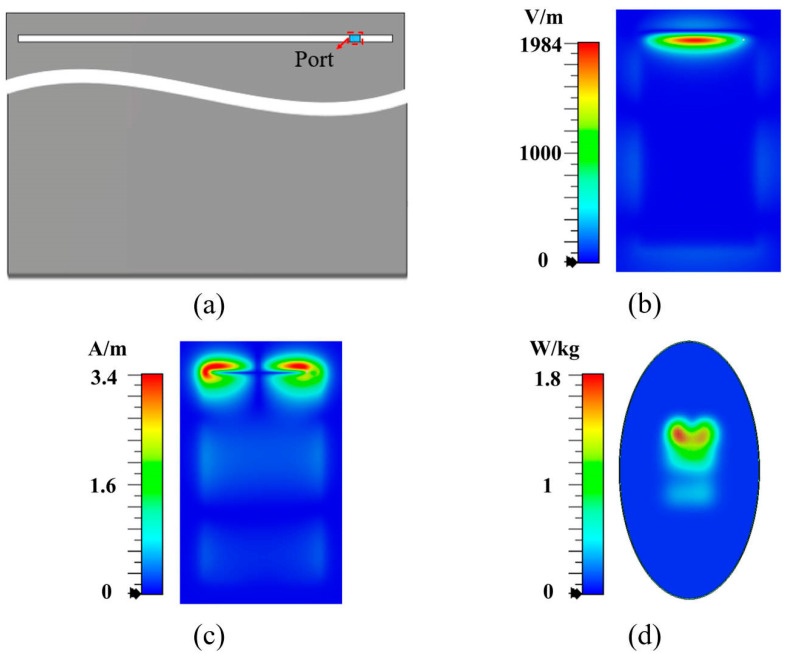
Slot antenna. (**a**) Overall structure, (**b**) tangential electric field, (**c**) tangential magnetic field, and (**d**) SAR distribution.

**Figure 2:**
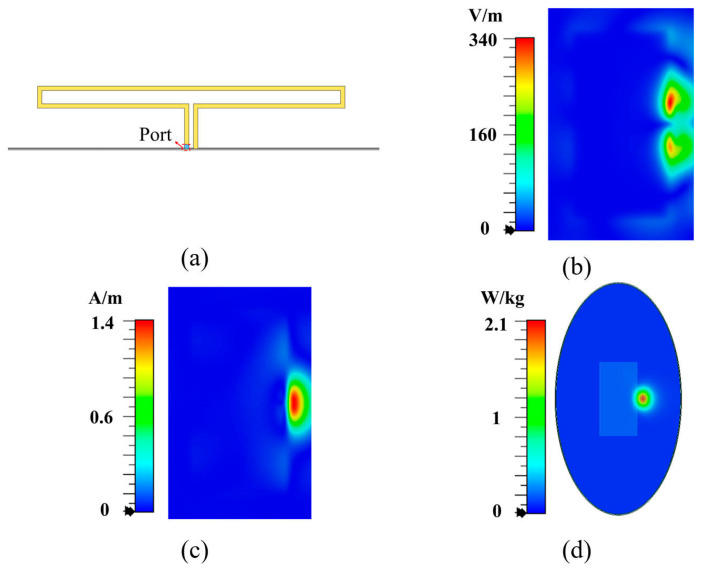
Loop antenna. (**a**) Overall structure, (**b**) tangential electric field, (**c**) tangential magnetic field, and (**d**) SAR distribution.

To clearly demonstrate the relationship between electromagnetic field hotspots and antenna SAR distribution, we used a slot terminal antenna operating at 0.5λ (shown in Figure 1a) and a loop antenna operating at 1λ (shown in Figure 2a) as two examples. The electromagnetic field distributions on the surfaces located 5 mm from the back side of antennas at their respective resonant frequencies are examined, and the SAR distributions of the antennas are also studied. Based on the IEC/IEEE International Standard [25], a simplified human body model is constructed, which is positioned 5 mm away from the back side of the antenna. As shown in Figure 1b,c, the tangential electric field of the 0.5λ slot antenna exhibits only one hotspot, while the tangential magnetic field presents two hotspots on each side of the slot, which is consistent with the SAR distribution shown in Figure 1d. Similarly, as shown in Figure 2b,c, the 1λ loop antenna exhibits two tangential electric field hotspots at both sides of the loop, while the tangential magnetic field shows one hotspot at the loop center, aligning with the SAR distribution in Figure 2d. Furthermore, we quantitatively analyzed the spatial correlation between SAR hotspots and tangential magnetic field hotspots. For the slot antenna in Figure 1, the Jaccard index reaches 0.71, indicating significant spatial overlap. Notably, for the loop antenna in Figure 2, the Jaccard index attains 0.93 with complete containment of tangential magnetic field hotspots within the SAR hotspots. These results indicate that the hotspot distribution of the tangential magnetic field exhibits a stronger correlation with the SAR distribution, compared to the tangential electric field.

This observation motivates us to analyze the intrinsic relationship between the tangential magnetic field and the SAR. Starting from the SAR definition [25]

(1) $$\mathrm{SAR}=\frac{d}{dt}\left(\frac{dW}{dm}\right)=\frac{d}{dt}\left(\frac{dW}{\rho dV}\right)=\frac{1}{\rho }\cdot \frac{dP}{dV}$$

where *ρ* is the density of human tissue, *V* is a given volume element in the human tissue, *W* is the incremental energy absorbed in the human tissue, and *P* is the power loss density in the human tissue.

**Figure 3:**
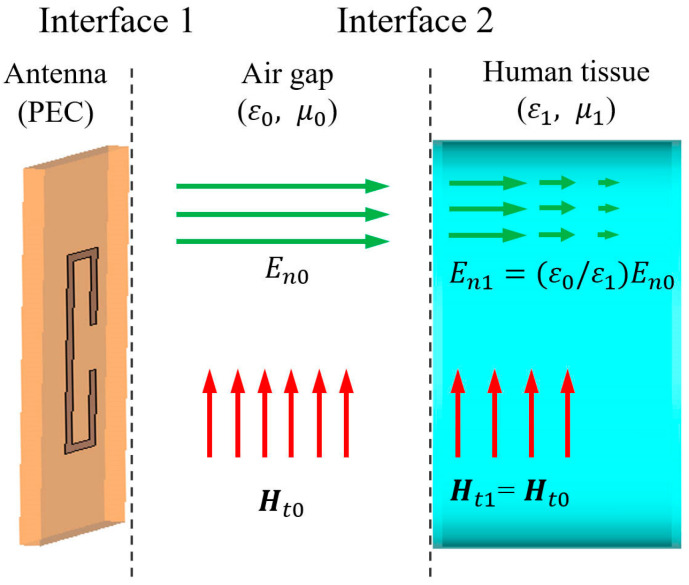
Field distributions on the antenna, the air gap, and the human tissue.

Based on the electromagnetic field boundary conditions, a three-layer model containing an antenna, an air gap and the human tissue is established [19], as shown in Figure 3. Considering the high conductivity of the antenna, we apply the perfect electric conductor boundary conditions at Interface 1 [26]. Then, only the normal electric field component $E_{n0}$ and tangential magnetic field component $H_{t0}$ exist on the antenna’s exterior surface. The tangential component of the electromagnetic field is a vector, so it is shown here in bold. Since the air gap between the antenna and the human tissue is very small, the field remains nearly constant across the air gap. The electromagnetic boundary conditions at Interface 2 can be expressed as Equation (2) [26].

(2) $$\begin{array}{l} E_{n1}=\left(\varepsilon_{0}/\varepsilon_{1}\right)E_{n0}\\ H_{n1}=\left(\mu_{0}/\mu_{1}\right)H_{n0}=0\\ E_{t1}=E_{t0}=0\\ H_{t1}=H_{t0} \end{array}$$

where $\varepsilon_{0}$ is the free-space permittivity, $\varepsilon_{1}$ is the permittivity of human tissue, and $\mu_{0}$ and $\mu_{1}$ represent the magnetic permeability of the air gap and human tissue, respectively.

Since $\varepsilon_{1}\gg 1$ [20], the normal electric field component demonstrates poor interfacial penetration into human tissue. On the other hand, due to the tangential electric field on the human tissue side being very small, it is difficult for the electric field to penetrate through the human body.

Considering the identical permeability of human tissue and air [19], it follows from Equation (2) that the magnetic field within tissue is predominantly tangential. This tangential component subsequently induces an internal electric field, as formulated in Equation (3) [27].

(3) $$E\approx \eta H_{t0}$$

where ***E*** is the electric field, and η is the wave impedance in the human tissue.

The power density of electromagnetic energy (d*P*) absorbed by a given volume element (d*V*) in the human body is expressed as Equation (4) [28].

(4) $$\frac{dP}{dt}=\frac{1}{2}\sigma {\left|E\right|}^{2}$$

where σ is the conductivity of human tissue. It follows from Equations (1), (3), and (4) that

(5) $$\mathrm{SAR}=\frac{1}{\rho }\cdot \frac{1}{2}\sigma {\left|\eta H_{t0}\right|}^{2}$$

As shown in Equation (5), the SAR value is positively correlated with the tangential magnetic field strength, which can be used to explain the correspondence between the location of the SAR hotspot of the antenna and the location of the tangential magnetic field strength.

### 2.2. SAR Reduction Mechanism Through Magnetic Field Homogenization

From the perspective of energy, the presence of multiple uniformly distributed SAR hotspots indicates that the electromagnetic energy absorbed by the human tissue is dispersed across different locations and distributed relatively evenly. Conversely, if only one SAR hotspot exists, the same radiation power would be concentrated at a single point, leading to a significant increase in the electromagnetic energy absorption at that specific location and consequently resulting in higher SAR values.

Given the dominant influence of tangential magnetic field components and multi-hotspot distributions on the SAR characteristics, we propose a magnetic field homogenization mechanism. The core concept involves redistributing the near-field tangential magnetic energy to suppress the localized peaks, thereby significantly reducing peak SAR values while maintaining constant total radiated power. This mechanism provides the theoretical foundation for the antenna design in Section 3, where the targeted structural optimization achieves the homogenized tangential magnetic field distributions on both the back and top planes. (These two planes correspond to the standardized SAR measurement planes defined in IEC/IEEE 62209-1528 [25].) Although Section 3 presents a single-band antenna design based on the magnetic-field homogenization mechanism, the proposed methodology remains applicable for the SAR reduction across multiple frequency bands.

## 3. Design of Low-SAR Antenna

**Figure 4:**
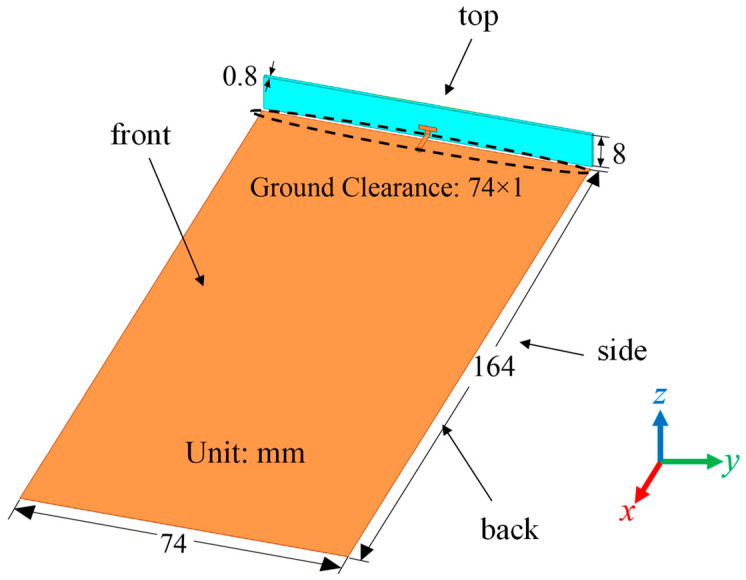
The shared foundational framework of three antennas.

**Figure 5:**
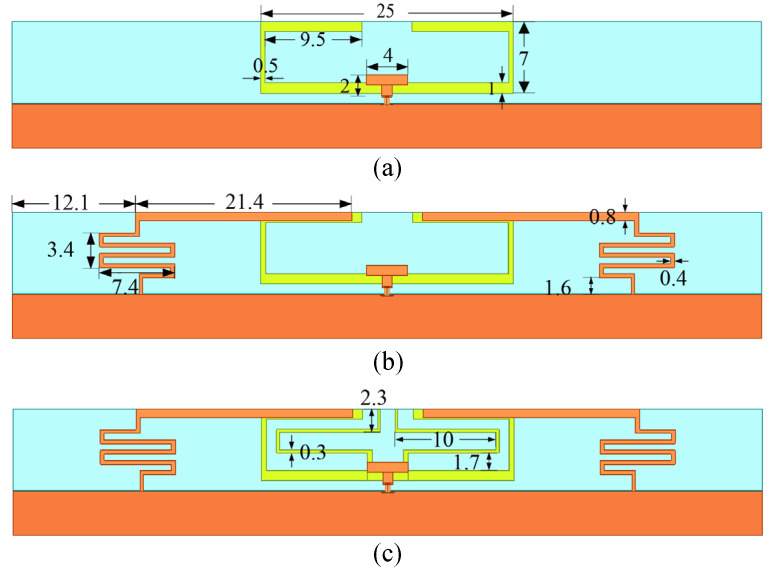
The main radiators of the three antennas. (**a**) Antenna A, (**b**) Antenna B, and (**c**) Antenna C.

By using the proposed mechanism, a low-SAR high-efficiency terminal antenna was designed. In order to explain its design principle, three antennas were designed first. Figure 4 illustrates the shared foundational framework of these three antennas, and their main radiators are presented in Figure 5. All the antennas adopt a ground clearance area with dimensions of 74 × 1 mm^2^. Figure 4 explicitly labels four planes adjacent to the main radiator of the antenna, i.e., top, back, front, and side planes. These planes serve as reference benchmarks for subsequent SAR analysis. The orange and yellow metal layers are printed on the inner and outer faces of the light blue FR4 dielectric substrate (*ε_r_* = 4.4, tan *δ* = 0.02, size: 74 × 8 × 0.8 mm^3^). All the simulation results are obtained using the CST Studio Suite 2023 software.

**Figure 6:**
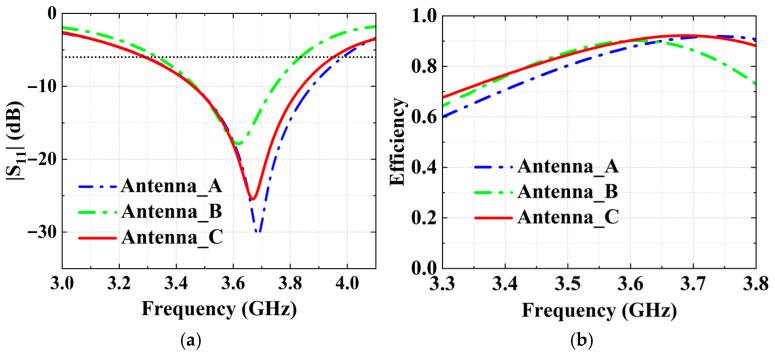
Simulation performance of these three antennas. (**a**) Reflection coefficients and (**b**) radiation efficiencies.

**Figure 7:**
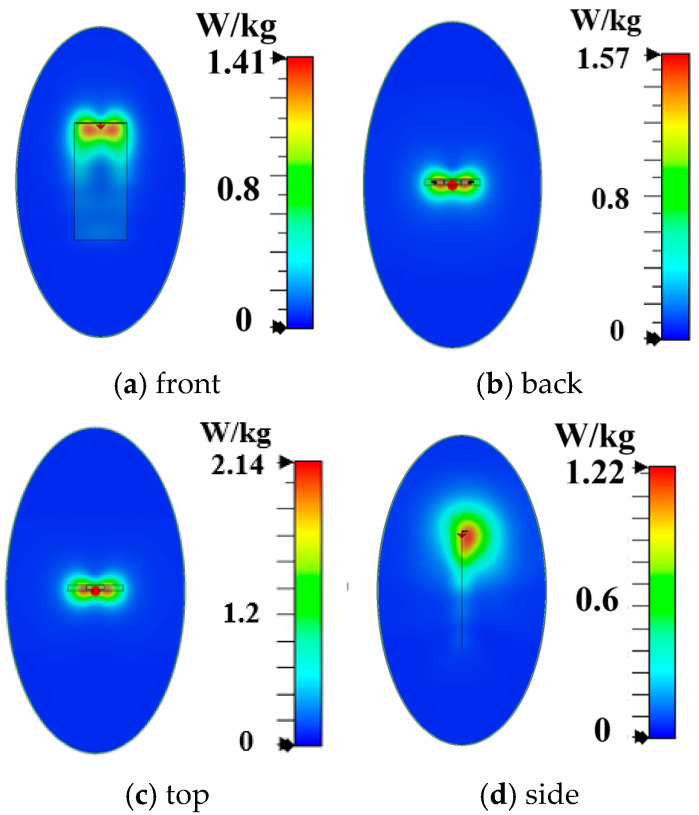
Simulated 10 g spatial-average body SAR values of Antenna A.

As shown in Figure 5a, the main radiator structure of Antenna A consists of two U-shaped monopoles and a T-shaped feeding structure. As shown in Figure 6a, its −6 dB reflection coefficient bandwidth covers the N78 band (3.3 GHz to 3.8 GHz). Simulated free-space radiation efficiency of Antenna A is presented in Figure 6b. Its radiation efficiencies are greater than 60% in the N78 band. The 10 g spatial-average body SARs of Antenna A at 3.6 GHz are illustrated in Figure 7. The SAR values are calculated when the antenna received power is set to 24 dBm and the distance between the antenna and the human tissue is set to 5 mm. During the SAR simulation, four planes (front, back, top, and side) of the antenna close to the human body are considered. As shown in Figure 7, the maximum peak SAR values in the top and back planes of Antenna A are higher. Therefore, we focus on reducing the SAR values on these two planes.

**Figure 8:**
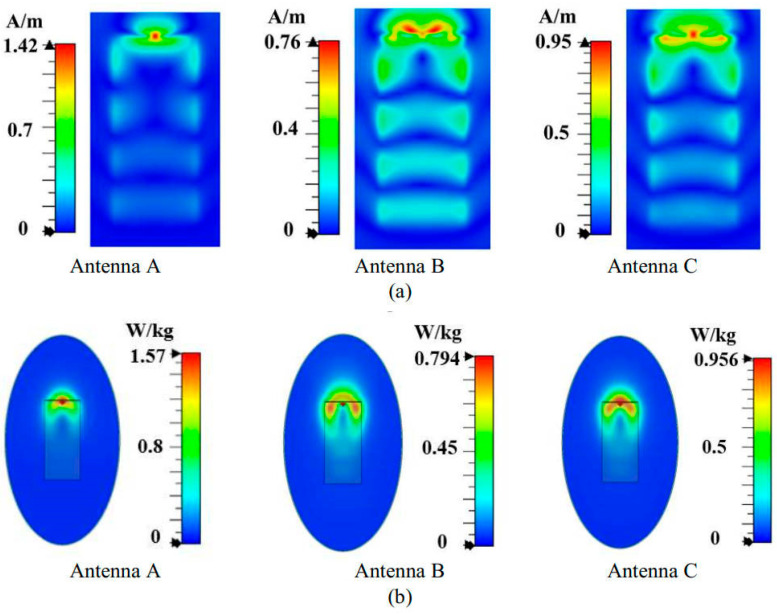
Performance of three antennas. (**a**) Maximum peaks of tangential magnetic fields in the plane located at 5 mm from the back side of the antenna, and (**b**) back SAR values.

The tangential magnetic field distribution is first analyzed on the plane located at 5 mm from Antenna A’s back side at 3.6 GHz. As shown in Figure 8a, the magnetic field distribution of Antenna A is characterized by a single hot spot at the center. To reduce the back SAR value, we considered introducing additional magnetic field hotspots at both ends of Antenna A to homogenize the magnetic field. Based on this concept, we structurally optimized Antenna A by symmetrically loading two bent inverted-L-shaped grounded branches on both sides, resulting in Antenna B, as illustrated in Figure 5b. The simulated results of Antenna B in Figure 8a, demonstrate that the modified structure successfully introduces new magnetic field hotspots on both sides, creating multiple red high field strength regions in the tangential magnetic field distribution and achieving a more uniform spatial distribution of the magnetic field. The optimized tangential magnetic field peak intensity is significantly reduced to 0.76 A/m. Further analysis of the SAR distribution in Figure 8b reveals that compared to Antenna A, the SAR hotspots (red regions) of the optimized Antenna B expand the distribution range toward both sides of the antenna. This more uniform energy distribution reduces the peak back SAR value to 0.76 W/kg, validating the effectiveness of the magnetic field homogenization mechanism.

**Figure 9:**
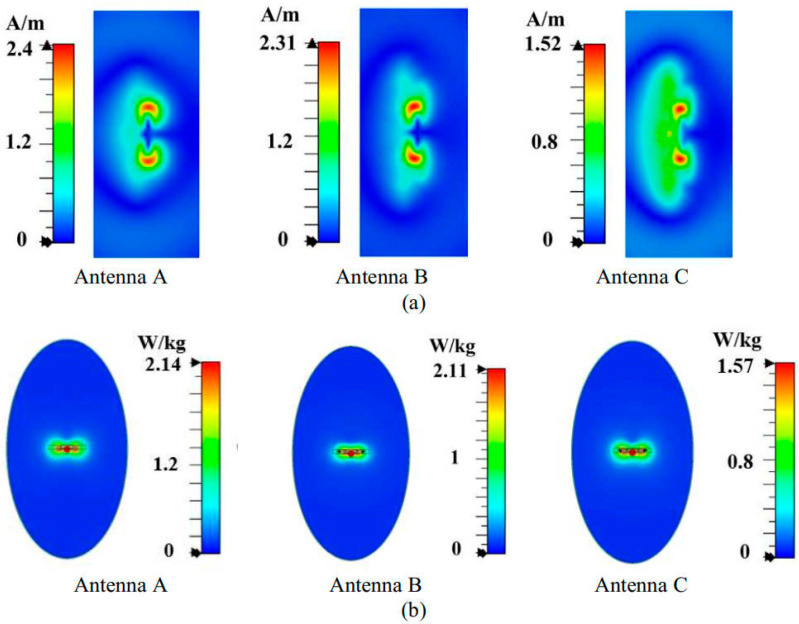
Performance of three antennas. (**a**) Maximum peaks of tangential magnetic fields in the plane located at 5 mm from the top side of the antenna, and (**b**) top SAR values.

However, Antenna B still faces the same problem as that of Antenna A. As shown in Figure 9a, the red region of the tangential component of the magnetic field from the top side of the antenna is almost unchanged, and the peak value is still high. We found that there is a blue weak magnetic field region in the middle of Antenna B. To address this phenomenon, we consider homogenization of the magnetic field by introducing an additional magnetic field hotspot in the central region. Implementation of this optimization involves structural modifications to Antenna B through the integration of a symmetrically folded monopole within the existing U-shaped monopole configuration, obtaining Antenna C, as shown in Figure 5c. As shown in Figure 9a, the improved antenna C effectively eliminates the blue weak magnetic field region in the middle and presents a yellow hotspot of stronger magnetic field, realizing the homogenization of the tangential magnetic field distribution. As shown in Figure 9b, the top SAR value of Antenna C is reduced to 1.57 W/kg.

Compared with Antenna A, Antenna C, based on the magnetic field homogenization mechanism, achieves a significant SAR improvement. Specifically, the back and top SAR values are reduced by 39% and 27%, respectively. These results fully validate the effectiveness of the magnetic field homogenization mechanism in reducing the SAR value of the antenna.

As shown in Figure 6b, the free-space efficiencies of Antenna C are higher than those of Antenna A at most frequencies in the N78 band, with the lowest efficiency exceeding 68%. This demonstrates that the magnetic field homogenization mechanism achieves the SAR reduction without compromising the antenna radiation efficiency.

## 4. Antenna Performance

**Figure 10:**
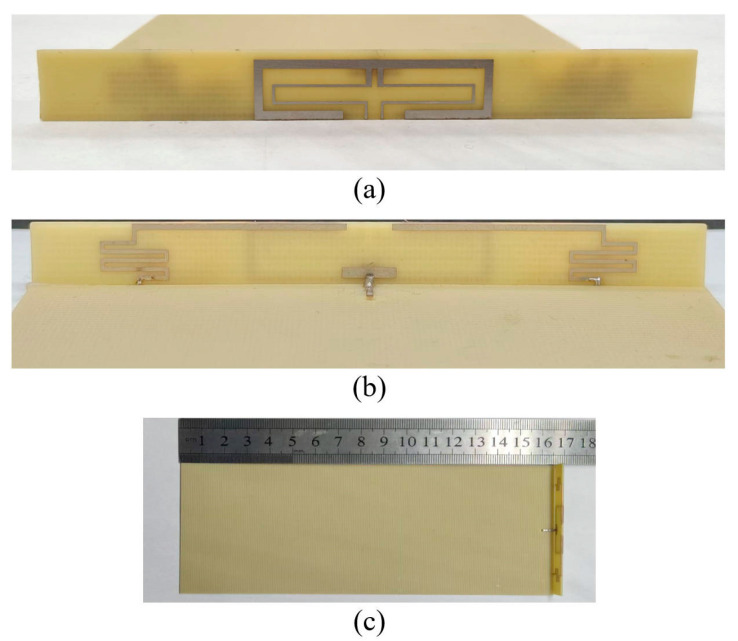
Photographs of the fabricated antenna. (**a**) Front side of the radiator, (**b**) back side of the radiator, and (**c**) overall structure of the antenna.

**Figure 11:**
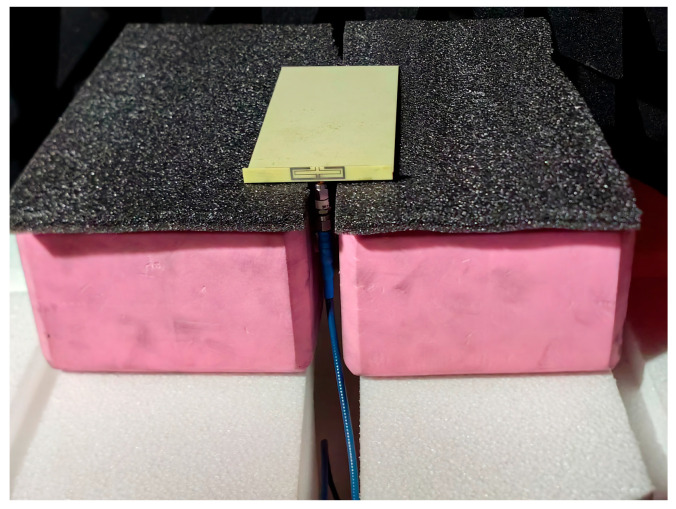
Antenna test environment.

**Figure 12:**
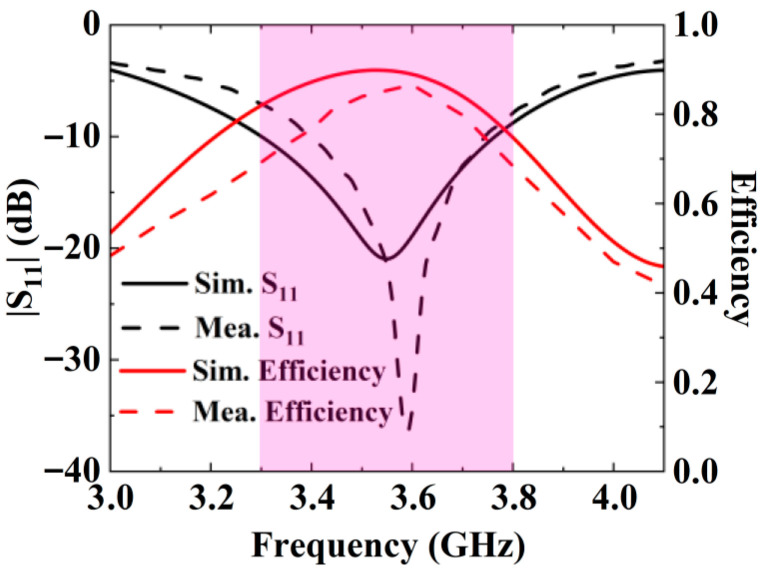
Simulated and measured results.

**Figure 13:**
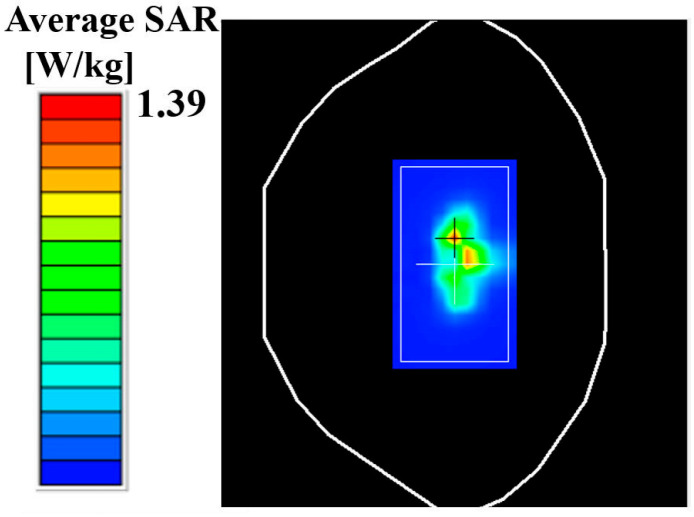
Measured 10 g average SARs.

**Figure 14:**
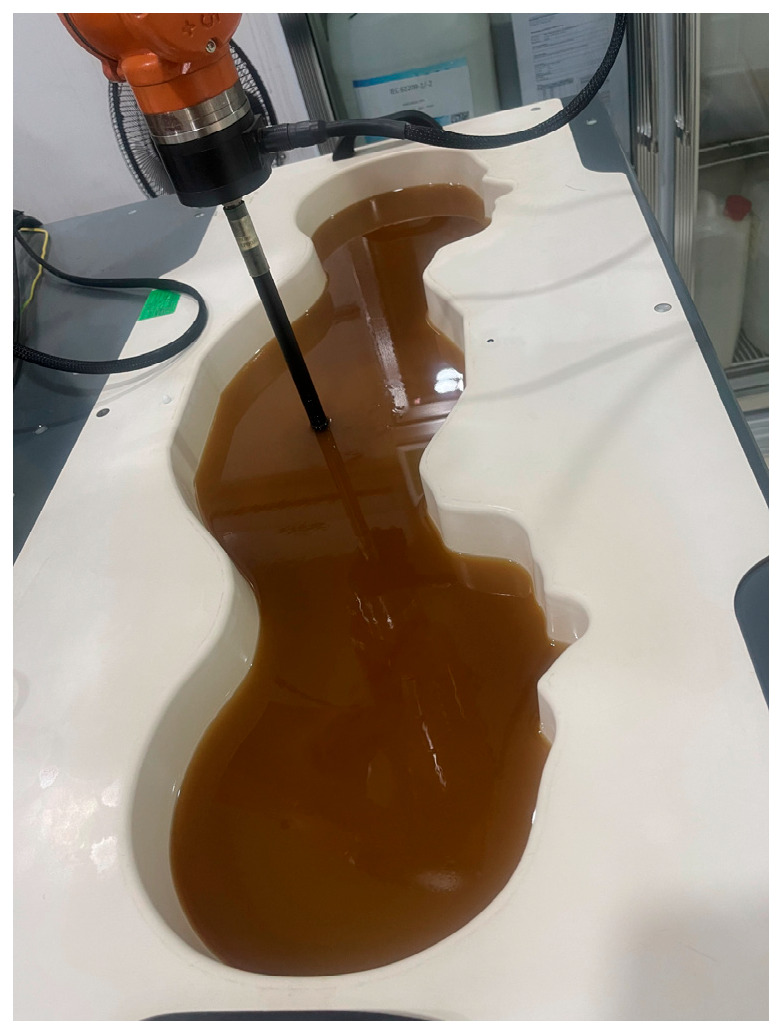
SAR test environment.

**Figure 15:**
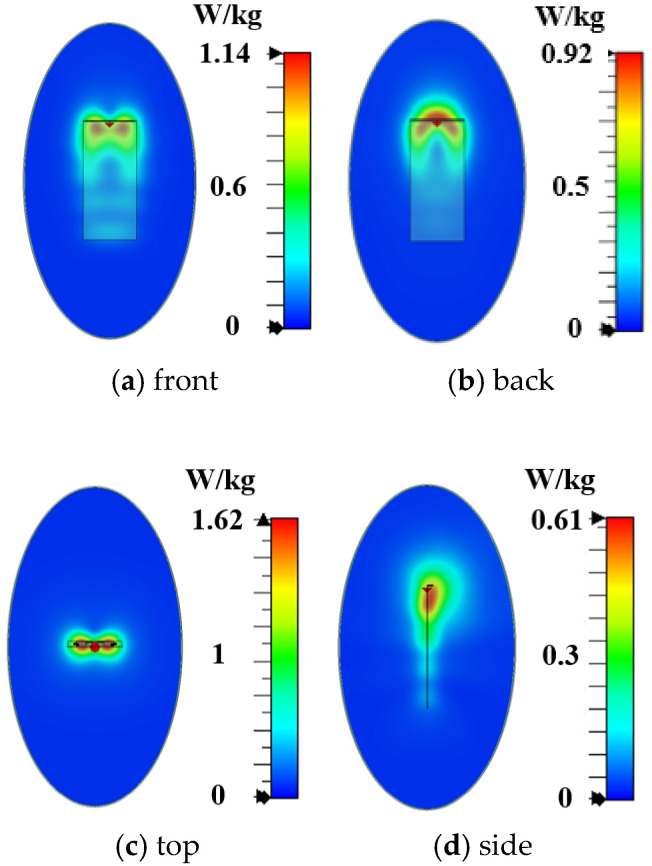
Simulated 10 g spatial-average body SAR values of the proposed antenna.

**Figure 16:**
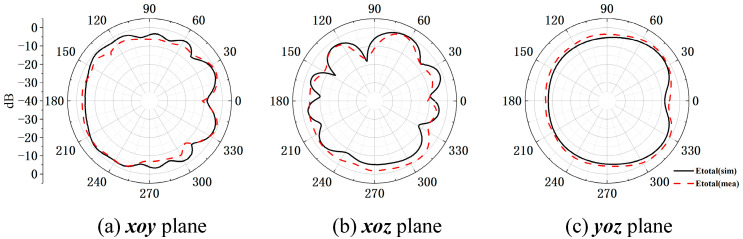
Measured and simulated 2D radiation patterns of the antenna at 3.6 GHz.

In order to verify the magnetic field homogenization mechanism, Antenna C loaded with the bottom dielectric substrate is fabricated and measured. Photographs of the fabricated antenna are shown in Figure 10. Dimensions of the antenna are modified due to the effect of the bottom dielectric substrate. The S parameters are measured using a Rohde and Schwarz ZVA 67 vector network analyzer (Rohde & Schwarz GmbH & Co. KG, Munich, Germany). The S-parameter test environment is shown in Figure 11. Simulated and measured S parameters of the proposed antenna are shown as black lines in Figure 12. The measured results show that the −6 dB reflection coefficient bandwidth of the proposed antenna covers 3.3 GHz to 3.8 GHz, which meets the 5G n77/n78 band requirements. The red lines in Figure 12 show the simulated and measured radiation efficiencies of the proposed antenna. Due to fabrication tolerances, measurement uncertainties, and cable losses, the measured radiation efficiencies are lower than the simulated values (simulated >78%, but measured >69% across the operating band), though their overall frequency-dependent trend aligns consistently with the measured S-parameters. Simulated efficiencies of the antenna in the operating band are higher than 78%, while its lowest measured efficiency is 69%. Due to the limitation of the test environment, we only measured the 10 g average top SAR distribution of the antenna at 3.6 GHz with the 24 dBm input power, as shown in Figure 13. The SAR values of the antenna are measured using a SATIMO measurement system (OneWave, Xi’an, China). The test environment for the SAR is shown in Figure 14. The 10 g average SAR of the proposed antenna is 1.39 W/kg. The reduced measured radiation efficiency diminishes energy coupling into human tissue, consequently lowering the measured SAR value compared to the simulated Top SAR in Figure 15. The simulated 10 g-spatial-average body SAR values of the proposed antenna at 3.6 GHz are provided in Figure 15. Figure 16 presents the measured and simulated 2D radiation patterns of the proposed antenna at 3.6 GHz. The radiation patterns are measured using a multi-probe near-field OTA antenna measurement system in accordance with CTIA test standards. As shown in Figure 16, the simulated results are in good agreement with the measured ones.

Comparison of the proposed low-SAR terminal antenna with some existing low-SAR antennas is shown in Table 1. The SAR values in Table 1 are the maximum peak back body SARs under an input power of 24 dBm to make a fair comparison. The values in parentheses are the normalized SARs to ensure the same radiated power (the efficiency is set to 22.5%) [20]. Obviously, the proposed antenna achieves relatively low SAR, simultaneously maintaining high radiation efficiencies.

## 5. Conclusions

In this paper, a mechanism based on the magnetic field homogenization is proposed to reduce the SAR values of the antenna. By adjusting the magnetic field distribution of the antenna, a uniform SAR hotspot distribution can be obtained, which significantly reduces the SAR performance. By using the SAR reduction mechanism, a low-SAR high-efficiency terminal antenna with an operating frequency band within 3.3 GHz to 3.8 GHz is designed. By adjusting the magnetic field distribution in two planes of the initial antenna with the maximum SAR values, 39% and 27% SAR reductions are achieved, respectively. The designed low-SAR terminal antenna is fabricated. The measured results show that the antenna has good efficiency and SAR performance, which can provide theoretical guidance for the antenna design to simultaneously realize high radiation efficiencies and low SAR.

## Figures and Tables

**Table 1:** Comparison of the proposed antenna with some existing SAR reduction antennas.

	Bandwidth (GHz)	SAR Reduction Mechanism	Back Body SAR (W/kg)	Simulated Efficiency (%)
[20]	0.83 to 1.04; 1.67 to 2.73	Reverse current	1.88 (1.06)	>34
[21]	3.4 to 3.6	Reverse mode current	1.01 (0.35)	>50
[24]	3.4 to 3.6	Total current equalization	1.67 (0.60)	>57
[29]	1.7 to 2.7	Uniform current	1.19 (0.31)	>67
[30]	1.7 to 2.7	without	1.53 (0.50)	>60
Prop.	3.3 to 3.8	Magnetic field homogenization	0.92 (0.23)	>78

## Data Availability

All data generated or analyzed during this study are included in this manuscript. There are no additional data or datasets beyond what is presented in the manuscript.
